# Middle East Respiratory Syndrome Coronavirus Transmission 

**DOI:** 10.3201/eid2602.190697

**Published:** 2020-02

**Authors:** Marie E. Killerby, Holly M. Biggs, Claire M. Midgley, Susan I. Gerber, John T. Watson

**Affiliations:** Centers for Disease Control and Prevention, Atlanta, Georgia, USA

**Keywords:** MERS-CoV, Middle East respiratory syndrome, coronavirus, emerging infectious disease, healthcare-associated transmission, dromedary, camel, epidemiology, viruses, zoonoses

## Abstract

Middle East respiratory syndrome coronavirus (MERS-CoV) infection causes a spectrum of respiratory illness, from asymptomatic to mild to fatal. MERS-CoV is transmitted sporadically from dromedary camels to humans and occasionally through human-to-human contact. Current epidemiologic evidence supports a major role in transmission for direct contact with live camels or humans with symptomatic MERS, but little evidence suggests the possibility of transmission from camel products or asymptomatic MERS cases. Because a proportion of case-patients do not report direct contact with camels or with persons who have symptomatic MERS, further research is needed to conclusively determine additional mechanisms of transmission, to inform public health practice, and to refine current precautionary recommendations.

Middle East respiratory syndrome (MERS) coronavirus (MERS-CoV) was first detected in Saudi Arabia in 2012 (*1*). To date, >2,400 cases globally have been reported to the World Health Organization (WHO), including >850 deaths (case fatality rate ≈35%) (*2*). Illness associated with MERS-CoV infection ranges from asymptomatic or mild upper respiratory illness to severe respiratory distress and death. 

MERS-CoV is a zoonotic virus, and dromedary camels are a reservoir host (*3*–*5*). Bats are a likely original reservoir; coronaviruses similar to MERS-CoV have been identified in bats (*6*), but epidemiologic evidence of their role in transmission is lacking. Infection of other livestock species with MERS-CoV is possible (*7*); however, attempts to inoculate goats, sheep, and horses with live MERS-CoV did not produce viral shedding (*8*), and no epidemiologic evidence has implicated any species other than dromedaries in transmission.

Sporadic zoonotic transmission from dromedaries has resulted in limited human-to-human transmission chains, usually in healthcare or household settings (*9*–*14*) (Figure). MERS-CoV human cases result from primary or secondary transmission. Primary transmission is classified as transmission not resulting from contact with a confirmed human MERS case-patient (*15*) and can result from zoonotic transmission from camels or from an unidentified source. Conzade et al. reported that, among cases classified as primary by the WHO, only 191 (54.9%) persons reported contact with dromedaries (*15*). Secondary transmission is classified as transmission resulting from contact with a human MERS case-patient, typically characterized as healthcare-associated or household-associated, as appropriate. However, many MERS case-patients have no reported exposure to a prior MERS patient or healthcare setting or to camels, meaning the source of infection is unknown. Among 1,125 laboratory-confirmed MERS-CoV cases reported to WHO during January 1, 2015–April 13, 2018, a total of 157 (14%) had unknown exposure (*15*).

**Figure:**
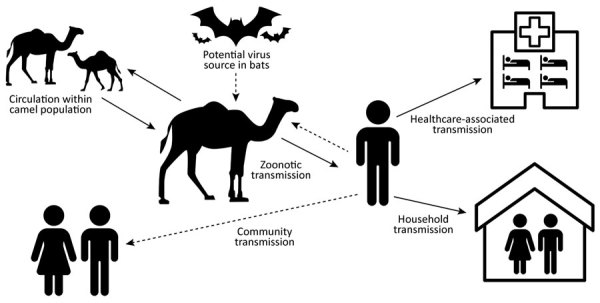
Summary of Middle East respiratory syndrome coronavirus transmission pathways. Solid lines indicate known transmission pathways; dashed lines indicate possible transmission pathways for which supporting evidence is limited or unknown.

Although broad categories of exposure are associated with transmission (e.g., exposure to camels or to healthcare facilities with ill patients), exact mechanisms of MERS-CoV transmission are not fully understood. Little direct epidemiologic evidence exists regarding transmission routes or the efficacy of interventions in reducing transmission. However, other potentially important factors, including detection of virus in different secretions, detection and survival of virus in the environment, and detection of virus in aerosols, lend support for the biological plausibility of certain transmission pathways. We summarize the available evidence regarding camel-to-camel, camel-to-human, and human-to-human transmission of MERS-CoV, including direct epidemiologic evidence and evidence supporting biologically plausible transmission routes.

## MERS-CoV in Camels

### Evidence for Infection of Camels

MERS-CoV infection in camels has been demonstrated through serologic investigations, molecular evidence using real-time reverse transcription PCR (rRT-PCR), and by virus isolation, as described in recent reviews (*16*,*17*). Geographically wide-ranging seroprevalence studies have identified MERS-CoV–specific antibodies in camels in countries across the Middle East and North, West, and East Africa, often with >90% seroprevalence in adult camels (*18*). Studies in many of these countries have shown molecular evidence of MERS-CoV RNA and isolation of infectious MERS-CoV in camels (*16*,*17*,*19*–*21*).

### Viral Shedding in Camels

In naturally or experimentally infected camels, MERS-CoV appears to cause an upper respiratory tract infection with or without symptoms, including nasal and lachrymal discharge, coughing, sneezing, elevated body temperature, and loss of appetite (*20*,*22*,*23*). In naturally infected camels, MERS-CoV RNA has been recovered most commonly from nasal swabs but also from fecal swabs, rectal swabs, and lung tissue (*20*,*24*). No evidence of viral RNA has been demonstrated in camel serum, blood, or urine using rRT-PCR (*25*,*26*). In experimentally infected camels, infectious virus and RNA was detected in nasal swab and oral samples but not in blood, serum, feces, or urine (*23*). MERS-CoV RNA has been detected in raw camel milk collected using traditional milking methods, including using a suckling calf as stimulus for milk letdown; presence of live virus was not evaluated (*27*). Virus RNA may therefore have been introduced via calf saliva or nasal secretions or fecal contamination. Experimentally introduced virus can survive in milk but did not survive when heat treated (*28*). It is also not known if the virus would remain viable in milk from seropositive dams when antibodies could be found in the milk.

These shedding data indicate that contact with camel nasal secretions, saliva, and respiratory droplets carry potential risk for camel-to-human or camel-to-camel transmission. Contact with nasal secretions can occur when directly handling live camels, and virus from camel nasal secretions can contaminate fomites in the environment (*29*). Although rRT-PCR evidence of MERS-CoV and genome fragments have been detected in air samples from a camel barn (*30*), no live virus was detected, and no epidemiologic study has implicated airborne transmission. Transmission following exposure to camel feces may be biologically plausible, although no epidemiologic evidence indicates the likelihood of such transmission. Similarly, although transmission following consumption of raw camel milk may be biologically plausible, epidemiologic studies have not consistently identified milk consumption as a unique risk factor for MERS-CoV infection or illness, independent of other direct or indirect camel exposures (*31*,*32*). No epidemiologic evidence supports transmission associated with camel urine or meat.

### Camel-to-Camel Transmission Dynamics

MERS-CoV RNA is detected most frequently in younger camels (*22*,*25*,*33*) but has been detected in camels >4 years of age (*22*). In a longitudinal study of a camel dairy herd, most calves became infected with MERS-CoV at 5–6 months of age, around the time maternal MERS-CoV antibodies wane. The calves then produced MERS-CoV antibodies by 11–12 months of age (*34*). In seroprevalence studies, camels <2 years of age demonstrated lower seroprevalence than camels >2 years of age (*25*,*35*). Across many countries, the seroprevalence of adult camels is >90% (*16*,*17*). Overall, these data suggest most camels are initially infected with MERS-CoV at <2 years of age. However, camels can shed virus despite preexisting MERS-CoV antibodies, suggesting that repeat infections are possible (*36*,*37*). Varying prevalence of MERS-CoV RNA in camels has been reported in different countries and settings, such as farms (*33*) and live animal markets (*38*). Risk for camel-to-camel or camel-to-human transmission may be influenced by crowding, mixing of camels from multiple sources, transportation, and characteristics of live animal markets (*39*). Phylogenetic modeling has provided supportive evidence that long-term MERS-CoV evolution has occurred exclusively in camels, with humans acting as a transient and usually terminal host (*40*).

## Zoonotic Transmission

### Evidence and Risk Factors for Zoonotic Transmission

Persons in Saudi Arabia with occupational exposure to camels demonstrated higher seroprevalence of MERS-CoV–specific antibodies (camel shepherds, 2.3%; slaughterhouse workers, 3.6%) compared with the general population (0.2%) (*41*). A case–control study of primary human cases and matched controls also showed that camel exposure was more likely among case-patients than matched controls (*31*). Further evidence supporting camel-to-human transmission includes identical or nearly identical MERS-CoV sequences found in camels and humans (*41*–*45*).

Occupational groups with frequent exposure to camels have been assessed through seroepidemiologic studies. In Qatar, a study of 9 seropositive and 43 matched seronegative camel workers showed that regular involvement in training and herding of camels, cleaning farm equipment, not handwashing before and after camel handling, and milking camels were associated with seropositivity (*46*). In a Saudi Arabia study of 30 camel workers in which 50% were seropositive for MERS-CoV, no association was identified between seropositivity and factors including age, smoking, handwashing after camel contact, consuming camel meat or milk, or specific occupation (camel truck driver, handler, or herder) (*47*). Neither investigation controlled for possible confounding risk factors (e.g., age or duration of exposure to camels). In Abu Dhabi, an investigation of 235 market and slaughterhouse workers showed that 17% were seropositive for MERS-CoV and that daily contact with camels or their waste, working as a camel salesman, and self-reported diabetes were risk factors for seropositivity (*32*). Among market workers in the same study, handling live camels and either cleaning equipment (e.g., halters, water troughs, etc.) or administering medications to camels were risk factors on multivariable analysis (*32*). These studies generally support the hypothesis that direct physical contact with camels is a risk factor for transmission, although cleaning equipment could also result in indirect transmission.

### Potential Seasonality of Human Cases

Previous findings suggest that MERS-CoV circulation among camels peaks in late winter through early summer (*22**,**26**,**48*). Initial MERS-CoV infection in camels is thought to occur at ≈6 months of age, after the typical winter calving season. Investigations have demonstrated different seasonal peaks for MERS-CoV infection in camels: November–January (*22*) and December–April (*48*) in Saudi Arabia, January–June in Egypt (*26*). A seasonal peak has been suggested to result in a corresponding peak in zoonotic transmission, and an April–July peak has been supported by phylogenetic modeling (*40*). However, camels have been found to be rRT-PCR positive for MERS-CoV throughout the year (*22*). Further investigations are needed to demonstrate seasonality of MERS-CoV infection in camels and link these patterns to seasonal peaks of zoonotic transmission.

### Zoonotic MERS-CoV Transmission outside the Arabian Peninsula

Despite evidence of circulating MERS-CoV in camels in North, East, and West Africa (*49*), limited evidence of human infection exists from Africa. In Kenya, no MERS-CoV antibodies were detected among 760 persons with occupational exposure to camels (*50*). In a separate study in Kenya, 2 seropositive adults who kept other livestock but not camels were identified among 1,122 persons who were predominantly without occupational exposure to camels (Appendix reference *51*). In Nigeria, no MERS-CoV antibodies were found in 261 slaughterhouse workers with exposure to camels (Appendix reference *52*). MERS-CoV sequences from camels in Africa phylogenetically cluster separately relative to camel and human MERS-CoV from the Arabian Peninsula, suggesting single or few introductions into Saudi Arabia and limited contact (*19*). Differences in virus growth have been shown between MERS-CoV strains isolated from West Africa and those isolated from the Middle East (*19*); relative to human and camel MERS-CoV from Saudi Arabia, virus isolates from Burkina Faso and Nigeria had lower virus replication competence in ex vivo cultures of human bronchus and lung. These findings may suggest regional variation in the potential for MERS-CoV replication in humans. Other factors contributing to the limited evidence of zoonotic MERS transmission in Africa may include differences between virus surveillance, human populations, camel populations, camel husbandry, and the type of human–camel interactions in these regions.

### Prevention of Zoonotic Transmission

WHO recommends that anyone presumed at higher risk for severe illness (e.g., persons who are older, have diabetes, or are immunocompromised) should avoid contact with camels and raw camel products (Appendix reference *53*). Although no evidence definitively links raw camel products with MERS-CoV infection, WHO presents these precautions in the context of considerable knowledge gaps surrounding MERS-CoV transmission. In addition, WHO recommends basic hygiene precautions for persons with occupational exposure to camels (Appendix reference *53*).

## Human-to-Human Transmission

After zoonotic introduction, human-to-human transmission of MERS-CoV can occur, but humans are considered transient or terminal hosts (*40*), with no evidence for sustained human-to-human community transmission. In addition to limited household transmission, large, explosive outbreaks in healthcare settings have been periodically documented. In South Korea in 2015, a single infected traveler returning from the Arabian Peninsula was linked to an outbreak of 186 cases, including 38 deaths (case-fatality rate 20%) (Appendix reference *54*). Multiple other healthcare-associated outbreaks have been described in Saudi Arabia (Appendix references *55*,*56*), Jordan (Appendix reference *57*), and United Arab Emirates (Appendix reference *58*). Healthcare transmission has also occurred outside the Arabian Peninsula from exported cases, including in the United Kingdom (Appendix reference *59*) and France (Appendix reference *60*). Given their size and scope, healthcare-associated outbreaks have provided most of the context for investigation of risk factors for human-to-human transmission.

### Viral Shedding in Humans

MERS-CoV shedding in humans appears to differ from the pattern of viral shedding in camels. In humans, MERS-CoV RNA and live virus have been detected in both upper (nasopharyngeal and oropharyngeal swab) and lower (sputum, tracheal aspirate, and bronchoalveolar lavage fluid) respiratory tract samples, although RNA levels are often higher in the lower respiratory tract (Appendix reference *61*). In humans, MERS-CoV is predominantly thought to infect the lower respiratory tract (Appendix reference *62*), where the MERS-CoV dipeptidyl peptidase-4 (DPP4) receptor predominates, in contrast to camels, where DPP4 is found predominantly in the upper respiratory tract (Appendix reference *63*). More severely ill patients typically have higher MERS-CoV RNA levels, as indicated by rRT-PCR cycle threshold (C_t_) values and more prolonged viral shedding (Appendix reference *64*). MERS-CoV RNA has been detected from the lower respiratory tract >1 month after illness onset (Appendix references *65*,*66*), and live virus has been isolated up to 25 days after symptom onset (Appendix reference *67*). RNA detection is prolonged in the respiratory tract of patients with diabetes mellitus, even when adjusting for illness severity (Appendix reference *66*). Among mildly ill patients, who might typically be isolated at home, viral RNA levels in the upper respiratory tract have been detected for several weeks (Appendix references *68*,*69*). Infectious virus has been isolated from the upper respiratory tract of a patient with mild symptoms (Appendix reference *68*), suggesting a potential for transmission among less severely ill patients. However, there is no definitive evidence of transmission from asymptomatic cases, and epidemiologic evidence suggests that transmission from mildly symptomatic or asymptomatic cases does not readily occur (Appendix reference *70*).

In humans, MERS-CoV RNA has been detected outside of the respiratory tract (Appendix references *66*,*71*,*72*). Viral RNA has been detected in the whole blood or serum of mildly or severely ill patients (Appendix references *66*,*72*) and in the urine of patients who subsequently died (Appendix reference *66*), although virus isolation attempts on urine samples (Appendix reference *66*) and serum (Appendix reference *71*) have been unsuccessful. MERS-CoV RNA has also been detected from the stool of mildly and severely ill patients (Appendix reference *66*). Subgenomic MERS-CoV RNA, an intermediate in the virus replication cycle, has been detected in stool, suggesting that MERS-CoV might replicate in the gastrointestinal tract (Appendix reference *73*); however, it is not clear if this contributes to pathogenesis or transmission.

### Reproduction Number and Attack Rates

The number of secondary cases resulting from a single initial case (reproduction number, R0) (Appendix reference *74*) ranges widely for MERS-CoV, e.g., from 8.1 in the South Korea outbreak, compared with an overall R0 of 0.45 in Saudi Arabia (Appendix reference *74*). Superspreading events, which generally describe a single MERS-CoV case epidemiologically linked to >5 subsequent cases, have been frequently described, particularly in healthcare-associated outbreaks (Appendix references *55*,*56*). R0 estimates, however, can vary depending on numerous biologic, sociobehavioral, and environmental factors, and must be interpreted with caution (Appendix reference *75*). Most studies estimating R0 across multiple areas, or at the end stage of an outbreak, result in estimates of R0<1, consistent with the knowledge that the virus does not continue to circulate in humans and that outbreaks are eventually contained. A wide range in published attack rates (the proportion of exposed persons who are infected) has also been reported (Appendix reference *74*).

### Transmission in Healthcare Facilities

Multiple studies have examined risk factors for MERS-CoV transmission in healthcare facilities. Higher viral loads (rRT-PCR C_t_ values) in respiratory tract samples have been linked to transmission risk (Appendix reference *76*). Kim et al. described heterogeneity of transmission in South Korea in 2015, where 22 of 186 cases were associated with further transmission of MERS-CoV and 5 superspreading events accounted for 150 of 186 cases (Appendix reference *54*). On multivariable analysis, transmission was associated with lower C_t_ values (indicating higher viral RNA load) and preisolation hospitalization or emergency department visits. Superspreading events were associated with a higher number of preisolation contacts, increased preisolation emergency room visits, and visiting multiple healthcare providers.

Alraddadi et al. studied risk factors for MERS-CoV infection in 20 healthcare workers in Saudi Arabia using serologic testing (Appendix reference *77*) and found that N95 respirator use among healthcare workers decreased the risk for seropositivity. Conversely, wearing a medical mask (as opposed to not wearing a medical mask) increased the risk for seropositivity, but this finding was observed in a small number of persons and was strongly correlated with not wearing an N95 respirator. All 20 healthcare workers had been in the same room or automobile or within 2 m of a MERS patient. This study provided evidence to suggest that aerosol transmission of MERS-CoV may be possible at close range, as seen with other respiratory viruses (e.g., influenza) spread primarily by droplet or contact transmission, particularly during aerosol-generating procedures. Having participated in infection control training specific to MERS-CoV was associated with a decreased risk for seropositivity; in healthcare workers in South Korea, a lack of personal protective equipment (PPE) use was more likely in MERS-CoV–infected healthcare workers than among exposed uninfected healthcare workers (Appendix reference *78*).

Studies have shown infection among persons without close and prolonged exposure to MERS case-patients during healthcare-associated outbreaks. In Jeddah during 2014, a total of 53 healthcare-associated cases were reported among hospitalized patients, of whom only 5 had documented presence in the same room as a MERS case-patient (Appendix reference *79*). Among the remaining healthcare-associated cases, many shared common treatment locations (e.g., dialysis unit) but denied being in the same room as a MERS case-patient. Similar observations were documented during a multihospital outbreak in Jordan in 2015, and anecdotal evidence suggested a potential role for fomite transmission associated with a common imaging table and portable echocardiogram machine (Appendix reference *80*).

MERS-CoV has been cultured from environmental objects, such as bed sheets, bedrails, intravenous fluid hangers, and radiograph devices, suggesting the potential for environmental transmission (Appendix reference *67*). Viral RNA has also been identified in air samples from the hospital rooms of MERS patients (Appendix reference *81*). MERS-CoV has also been shown to be relatively stable in the environment under various conditions (Appendix reference *82*), supporting the possibility of fomite transmission, although definitive epidemiologic evidence implicating fomite or aerosol transmission is lacking.

### Prevention of Healthcare-Associated Transmission

Studies have described delays in case recognition and establishment of infection control precautions as factors in healthcare-associated transmission (Appendix references *54*,*56*,*79*). Triage practices that result in rapid isolation of suspected MERS case-patients and application of transmission-based precautions can decrease opportunities for early transmission. However, implementing triage procedures to quickly identify potential MERS cases in areas with local MERS-CoV transmission (e.g., Arabian Peninsula) is challenging because signs and symptoms are often nonspecific (Appendix reference *83*). In addition, complications or exacerbations of concurrent conditions, including chronic kidney or heart disease, can manifest with acute or worsening respiratory symptoms that delay suspicion for MERS. Patient crowding has been associated with transmission in healthcare facilities, particularly in emergency departments (Appendix references *54*,*79*). In multiple outbreaks, inconsistent PPE use has been reported as contributing to transmission (Appendix references *56,58*), and transmission to healthcare personnel despite reported use of appropriate PPE (Appendix references *56*,*78*) suggests that improper PPE use may contribute to transmission. Transmission risk may be particularly high during aerosol-generating procedures, in which large quantities of virus might be aerosolized. 

### Household Transmission

Human-to-human transmission of MERS-CoV has been reported among household contacts. Drosten et al. described 12 probable cases among 280 household contacts of 26 index case-patients (*13*). Arwady et al. investigated MERS-CoV infections in an extended family of 79 relatives, of whom 19 (24%) tested positive for MERS-CoV by rRT-PCR or serology (Appendix reference *84*). Risk factors for acquisition included sleeping in an index case-patient’s room and touching their secretions. A study of MERS-CoV infection in a group of expatriate women housed in a dormitory in Riyadh, Saudi Arabia, showed an overall infection attack rate of 2.7% (Appendix reference *85*). Risk factors for infection include direct contact with a confirmed case-patient and sharing a room with a confirmed case-patient; a protective factor was having an air conditioner in the bedroom. However, transmission among household contacts is variable; Hosani et al. found that none of 105 household contacts of 34 MERS-CoV case-patients showed antibodies to MERS-CoV (Appendix reference *70*). Among those 34 patients, 31 were asymptomatic or mildly symptomatic, suggesting a lower risk for transmission among this group.

### Viral Factors Affecting Human-to-Human Transmission

No evidence has been reported that mutations or recombinations in MERS-CoV have led to changes in human-to-human transmission. Recombination has been documented among coronaviruses (Appendix reference *86*) and has been linked to increasing pathogenicity in strains of other animal RNA viruses (Appendix reference *87*). Circulation of recombinant MERS-CoV has beenoc described in Saudi Arabia in camels (*48*) and humans (Appendix references *88,89*) but no substantial change in human epidemiology was seen with this circulating variant (Appendix reference *89*). Deletion variants of MERS-CoV were identified in humans in Jordan (Appendix reference *57*), also without notable changes in epidemiology (Appendix reference *80*).

## Conclusions

In areas in which MERS-CoV actively circulates among camels, human cases can result from zoonotic transmission. In these areas, close contact with camels (e.g., handling or training) is an identified risk factor for infection. Direct or indirect contact with nasal secretions probably plays a role. Given limited knowl­edge of mechanisms of MERS-CoV transmission, current precautions to prevent zoonotic transmission, such as recommendations to avoid consumption of raw cam­el milk and meat, are prudent despite the lack of epidemiologic evidence linking these exposures to MERS-CoV infec­tion. Such precautionary recommendations, while appropriate in the context of limited knowledge, should not be interpreted as evidence of an epidemiologic association with MERS-CoV transmission.

Human-to-human transmission of MERS-CoV most frequently occurs following close contact with MERS patients, predominantly in healthcare settings and less frequently in household settings. Specifically, contact with respiratory secretions, whether through direct contact or through aerosolization of secretions during aerosol-generating procedures, plays a role in transmission. Virus isolation from fomites suggests the potential for alternative mechanisms of transmission, but direct epidemiologic evidence is lacking. Although MERS-CoV has been isolated from a mildly ill case-patient, available evidence is not sufficient to conclusively state that asymptomatic patients play an appreciable role in MERS-CoV transmission. Given the knowledge gaps surrounding transmission from asymptomatic patients, WHO recommendations state “until more is known, asymptomatic RT-PCR positive persons should be isolated, followed up daily for development of any symptoms, and tested at least weekly—or earlier, if symptoms develop—for MERS-CoV” (Appendix reference *90*). Available evidence supports published Centers for Disease Control and Prevention guidance for infection prevention and control for hospitalized MERS patients (Appendix reference *91*).

Large, explosive MERS-CoV outbreaks have repeatedly resulted in devastating impacts on health systems and in settings where transmission most frequently occurs. Sporadic community cases continue to be reported, and a small but consistent proportion of MERS cases have no camel, healthcare, or MERS-CoV exposure. Continuous epidemiologic and virologic monitoring is required to determine other exposures resulting in transmission and to assess for the possibility of improved virus fitness and adaptation. Until additional evidence is available to further refine recommendations to prevent MERS-CoV transmission, continued use of existing precautionary recommendations is necessary.

Appendix Additional information about Middle East respiratory syndrome coronavirus transmission.
