# Supplementary material for: Middle East Respiratory Syndrome Coronavirus Transmission
Source: Emerg Infect Dis. 2020 Feb;26(2):191–8. doi: 10.3201/eid2602.190697 (PMC6986839; doi:10.3201/eid2602.190697)
Supplement: Appendix — Additional information about Middle East respiratory syndrome coronavirus transmission. [file 19-0697-Techapp-s1.pdf]

Article DOI: https://doi.org/10.3201/eid2602.190697

Middle East Respiratory Syndrome Coronavirus Transmission

Appendix

Additional References

51. Liljander A, Meyer B, Jores J, Müller MA, Lattwein E, Njeru I, et al. MERS-CoV antibodies in humans, Africa, 2013–2014. Emerg Infect Dis. 2016;22:1086–9. PubMed https://doi.org/10.3201/eid2206.160064

52. So RT, Perera RA, Oladipo JO, Chu DK, Kuranga SA, Chan KH, et al. Lack of serological evidence of Middle East respiratory syndrome coronavirus infection in virus exposed camel abattoir workers in Nigeria, 2016. Euro Surveill. 2018;23. PubMed https://doi.org/10.2807/1560-7917.ES.2018.23.32.1800175

53. World Health Organization. WHO MERS global summary and assessment of risk. 2018 [cited 2019 Nov 1]. https://www.who.int/csr/disease/coronavirus_infections/risk-assessment-august-2018.pdf

54. Kim SW, Park JW, Jung H-D, Yang J-S, Park Y-S, Lee C, et al. Risk factors for transmission of Middle East respiratory syndrome coronavirus infection during the 2015 outbreak in South Korea. Clin Infect Dis. 2017;64:551–7. PubMed

55. Oboho IK, Tomczyk SM, Al-Asmari AM, Banjar AA, Al-Mugti H, Aloraini MS, et al. 2014 MERS-CoV outbreak in Jeddah—a link to health care facilities. N Engl J Med. 2015;372:846–54. PubMed https://doi.org/10.1056/NEJMoa1408636

56. Alanazi KH, Killerby ME, Biggs HM, Abedi GR, Jokhdar H, Alsharef AA, et al. Scope and extent of healthcare-associated Middle East respiratory syndrome coronavirus transmission during two contemporaneous outbreaks in Riyadh, Saudi Arabia, 2017. Infect Control Hosp Epidemiol. 2019;40:79–88. PubMed https://doi.org/10.1017/ice.2018.290

57. Lamers MM, Raj VS, Shafei M, Ali SS, Abdallh SM, Gazo M, et al. Deletion variants of Middle East respiratory syndrome coronavirus from humans, Jordan, 2015. Emerg Infect Dis. 2016;22:716–9. PubMed https://doi.org/10.3201/eid2204.152065

58. Hunter JC, Nguyen D, Aden B, Al Bandar Z, Al Dhaheri W, Abu Elkheir K, et al. Transmission of Middle East respiratory syndrome coronavirus infections in healthcare settings, Abu Dhabi. Emerg Infect Dis. 2016;22:647–56. PubMed https://doi.org/10.3201/eid2204.151615

59. Health Protection Agency (HPA) UK Novel Coronavirus Investigation team. Evidence of person-to-person transmission within a family cluster of novel coronavirus infections, United Kingdom, February 2013. Euro Surveill. 2013;18:20427. PubMed

60. Mailles A, Blanckaert K, Chaud P, van der Werf S, Lina B, Caro V, et al. First cases of Middle East respiratory syndrome coronavirus (MERS-CoV) infections in France, investigations and implications for the prevention of human-to-human transmission, France, May 2013. Euro Surveill. 2013;18:20502.

61. Memish ZA, Al-Tawfiq JA, Makhdoom HQ, Assiri A, Alhakeem RF, Albarrak A, et al. Respiratory tract samples, viral load, and genome fraction yield in patients with Middle East respiratory syndrome. J Infect Dis. 2014;210:1590–4. PubMed https://doi.org/10.1093/infdis/jiu292

62. van den Brand JM, Smits SL, Haagmans BL. Pathogenesis of Middle East respiratory syndrome coronavirus. J Pathol. 2015;235:175–84. PubMed https://doi.org/10.1002/path.4458

63. Widagdo W, Raj VS, Schipper D, Kolijn K, van Leenders GJLH, Bosch BJ, et al. Differential expression of the Middle East respiratory syndrome coronavirus receptor in the upper respiratory tracts of humans and dromedary camels. J Virol. 2016;90:4838–42. PubMed https://doi.org/10.1128/JVI.02994-15

64. Oh MD, Park WB, Choe PG, Choi S-J, Kim J-I, Chae J, et al. Viral load kinetics of MERS coronavirus infection. N Engl J Med. 2016;375:1303–5. PubMed https://doi.org/10.1056/NEJMc1511695

65. Poissy J, Goffard A, Parmentier-Decrucq E, Favory R, Kauv M, Kipnis E, et al.; MERS-CoV Biology Group. Kinetics and pattern of viral excretion in biological specimens of two MERS-CoV cases. J Clin Virol. 2014;61:275–8. PubMed https://doi.org/10.1016/j.jcv.2014.07.002

66. Al-Abdely HM, Midgley CM, Alkhamis AM, Abedi GR, Lu X, Binder AM, et al. Middle East respiratory syndrome coronavirus infection dynamics and antibody responses among clinically diverse patients, Saudi Arabia. Emerg Infect Dis. 2019;25:753–66. PubMed https://doi.org/10.3201/eid2504.181595

67. Bin SY, Heo JY, Song M-S, Lee J, Kim E-H, Park S-J, et al. Environmental contamination and viral shedding in MERS patients during MERS-CoV outbreak in South Korea. Clin Infect Dis. 2016;62:755–60. PubMed https://doi.org/10.1093/cid/civ1020

68. Al-Abdely HM, Midgley CM, Alkhamis AM, Abedi GR, Tamin A, Binder AM, et al., editors. Infectious MERS-CoV isolated from a mildly ill patient, Saudi Arabia. Open Forum Infect Dis. 2018;5:ofy111.

69. Al-Gethamy M, Corman VM, Hussain R, Al-Tawfiq JA, Drosten C, Memish ZA. A case of long-term excretion and subclinical infection with MERS-coronavirus in a health care worker. Clin Infect Dis. 2015;60:973–4. https://doi.org/10.1093/cid/ciu1135.

70. Al Hosani FI, Kim L, Khudhair A, Pham H, Al Mulla M, Al Bandar Z, et al. Serologic follow-up of Middle East respiratory syndrome coronavirus cases and contacts-Abu Dhabi, United Arab Emirates. Clin Infect Dis. 2019;68:409–18. PubMed https://doi.org/10.1093/cid/ciy503

71. Corman VM, Albarrak AM, Omrani AS, Albarrak MM, Farah ME, Almasri M, et al. Viral shedding and antibody response in 37 patients with Middle East respiratory syndrome coronavirus infection. Clin Infect Dis. 2016;62:477–83. PubMed

72. Oh MD, Park WB, Choe PG, Choi SJ, Kim JI, Chae J, et al. Viral load kinetics of MERS coronavirus infection. N Engl J Med. 2016;375:1303–5. PubMed https://doi.org/10.1056/NEJMc1511695

73. Zhou J, Li C, Zhao G, Chu H, Wang D, Yan HH-N, et al. Human intestinal tract serves as an alternative infection route for Middle East respiratory syndrome coronavirus. Science Advances. 2017;3:eaao4966.

74. Park J-E, Jung S, Kim A, Park J-E. MERS transmission and risk factors: a systematic review. BMC Public Health. 2018;18:574. PubMed https://doi.org/10.1186/s12889-018-5484-8

75. Delamater PL, Street EJ, Leslie TF, Yang YT, Jacobsen KH. Complexity of the basic reproduction number (R_0). Emerg Infect Dis. 2019;25:1–4. PubMed https://doi.org/10.3201/eid2501.171901

76. Feikin DR, Alraddadi B, Qutub M, Shabouni O, Curns A, Oboho IK, et al. Association of higher MERS-CoV virus load with severe disease and death, Saudi Arabia, 2014. Emerg Infect Dis. 2015;21:2029–35. PubMed https://doi.org/10.3201/eid2111.150764

77. Alraddadi BM, Al-Salmi HS, Jacobs-Slifka K, Slayton RB, Estivariz CF, Geller AI, et al. Risk factors for Middle East respiratory syndrome coronavirus infection among healthcare personnel. Emerg Infect Dis. 2016;22:1915–20. PubMed https://doi.org/10.3201/eid2211.160920

78. Kim C-J, Choi WS, Jung Y, Kiem S, Seol HY, Woo HJ, et al. Surveillance of the Middle East respiratory syndrome (MERS) coronavirus (CoV) infection in healthcare workers after contact with confirmed MERS patients: incidence and risk factors of MERS-CoV seropositivity. Clin Microbiol Infect. 2016;22:880–6. PubMed https://doi.org/10.1016/j.cmi.2016.07.017

79. Hastings DL, Tokars JI, Abdel Aziz IZ, Alkhaldi KZ, Bensadek AT, Alraddadi BM, et al. Outbreak of Middle East respiratory syndrome at tertiary care hospital, Jeddah, Saudi Arabia, 2014. Emerg Infect Dis. 2016;22:794–801. PubMed https://doi.org/10.3201/eid2205.151797

80. Payne DC, Biggs HM, Al-Abdallat MM, Alqasrawi S, Lu X, Abedi GR, et al. Multihospital outbreak of a Middle East respiratory syndrome coronavirus deletion variant, Jordan: a molecular, serologic, and epidemiologic investigation. Open Forum Infect Dis. 2018;5:ofy095

81. Kim S-H, Chang SY, Sung M, Park JH, Bin Kim H, Lee H, et al. Extensive viable Middle East respiratory syndrome (MERS) coronavirus contamination in air and surrounding environment in MERS isolation wards. Clin Infect Dis. 2016;63:363–9. PubMed https://doi.org/10.1093/cid/ciw239

82. van Doremalen N, Bushmaker T, Munster VJ. Stability of Middle East respiratory syndrome coronavirus (MERS-CoV) under different environmental conditions. Euro Surveill. 2013;18:20590. PubMed https://doi.org/10.2807/1560-7917.ES2013.18.38.20590

83. Alfaraj SH, Al-Tawfiq JA, Gautret P, Alenazi MG, Asiri AY, Memish ZA. Evaluation of visual triage for screening of Middle East respiratory syndrome coronavirus patients. New Microbes New Infect. 2018;26:49–52. PubMed https://doi.org/10.1016/j.nmni.2018.08.008

84. Arwady MA, Alraddadi B, Basler C, Azhar EI, Abuelzein E, Sindy AI, et al. Middle East respiratory syndrome coronavirus transmission in extended family, Saudi Arabia, 2014. Emerg Infect Dis. 2016;22:1395–402. PubMed https://doi.org/10.3201/eid2208.152015

85. Van Kerkhove MD, Alaswad S, Assiri A, Perera RAPM, Peiris M, El Bushra HE, et al. Transmissibility of MERS-CoV infection in closed setting, Riyadh, Saudi Arabia, 2015. Emerg Infect Dis. 2019;25:1802–9. PubMed https://doi.org/10.3201/eid2510.190130

86. Graham RL, Baric RS. Recombination, reservoirs, and the modular spike: mechanisms of coronavirus cross-species transmission. J Virol. 2010;84:3134–46. PubMed https://doi.org/10.1128/JVI.01394-09

87. Zhao K, Ye C, Chang X-B, Jiang C-G, Wang S-J, Cai X-H, et al. Importation and recombination are responsible for the latest emergence of highly pathogenic PRRSV in China. J Virol. 2015:89:10712–16.

88. Assiri AM, Biggs HM, Abedi GR, Lu X, Bin Saeed A, Abdalla O, et al.. Increase in Middle East respiratory syndrome-coronavirus cases in Saudi Arabia linked to hospital outbreak with continued circulation of recombinant virus, July 1–August 31, 2015. Open Forum Infect Dis. 2016;3:ofw165

89. Assiri AM, Midgley CM, Abedi GR, Bin Saeed A, Almasri MM, Lu X, et al. Epidemiology of a novel recombinant Middle East respiratory syndrome coronavirus in humans in Saudi Arabia. J Infect Dis. 2016;214:712–21. PubMed https://doi.org/10.1093/infdis/jiw236

90. World Health Organization. Management of asymptomatic persons who are RT-PCR positive for Middle East respiratory syndrome coronavirus (MERS-CoV). Interim guidance. 3 January 2018 [cited 2019 Nov 1]. https://apps.who.int/iris/bitstream/handle/10665/180973/WHO_MERS_IPC_15.2_eng.pdf

91. Centers for Disease Control and Prevention. Interim infection prevention and control recommendations for hospitalized patients with Middle East respiratory syndrome coronavirus (MERS-CoV). 2015 [cited 2019 Nov 1]. https://www.cdc.gov/coronavirus/mers/infection-prevention-control.html
